# Video clips of the Mediterranean Diet on YouTube ^TM^: A social Media Content Analysis

**DOI:** 10.1177/08901171221132113

**Published:** 2022-10-03

**Authors:** Nada Benajiba, Maha Alhomidi, Fahdah Alsunaid, Aljawharah Alabdulkarim, Elizabeth Dodge, Enmanuel A. Chavarria, Basil H. Aboul-Enein

**Affiliations:** 1 Joint Research Unit in Nutrition and Food, RDC-Nutrition AFRA/IAEA, 108308 Ibn Tofail University-CNESTEN, Kenitra, Morocco; 2 Clinical Nutrition Program, Department of Health Sciences, College of Health and Rehabilitation, 112893 Princess Nourah Bint Abdulrahman University, Riyadh, Saudi Arabia; 3 College of Graduate & Professional Studies, University of New England, Portland, ME, USA; 4 Department of Behavioral, Social, and Health Education Sciences, Rollins School of Public Health, 25798 Emory University, Atlanta, GA, USA; 5 Department of Health Science, Johnson & Wales University, College of Health & Wellness, Providence, RI, USA; 6 Faculty of Public Health and Policy, 4906 London School of Hygiene & Tropical Medicine, London, UK

**Keywords:** social media, mediterranean diet, youtube, internet

## Abstract

**Purpose:**

The present study conducted a social media content analysis on videos describing the Mediterranean Diet (MedDiet) posted onYouTube.

**Setting:**

YouTube TM online video sharing and social media platform.

**Method:**

Three independent content experts evaluated 141 YouTube videos on the MedDiet in August 2020 utilizing standard rubric and protocol. Data abstracted include media source(s) of posted videos, and viewer exposure/engagement metrics. Information quality was measured by each content expert independently through use of the DISCERN instrument, a 16-item tool designed to assess reliability, dependability, and trustworthiness of an online source, scores were then aggregated for analysis.

**Results:**

A majority of videos (n = 102, 72.3%) were educational in nature. A third of videos were less clear and less credible on information presented (n = 46, 32.6%). Most videos were posted by an individual (n = 79, 56%), and the majority of videos were rated as medium quality (n = 88, 62.4%). Overall level of user engagement as measured by number of “likes,” “dislikes,” and user comments varied widely across all sources of media. Exploratory correlation analysis suggests that the number of a video’s views, comments, likes, and dislikes are not correlated with quality.

**Conclusion:**

Study findings suggest that MedDiet health promotion and education via YouTube has the potential to reach and inform clients; however, existing video content and quality varies significantly. Future intervention research focused on MedDiet should further examine possible predictors of high quality MedDiet content utilizing diverse online video sharing platforms.

## Introduction

Globally, the number of Internet users has increased dramatically in the past decade, where, approximately two-thirds of the world’s population now has Internet access.^1^ Currently, people with Internet access are able to produce and share health information via social media (Web 2.0); this is in stark contrast to the early Internet where people were only able to retrieve information.^2,3^ Social media is considered a highly important tool for communicating health information.^4^ Coincidently, online health information (OHI) happens to be one of the most popular activities completed online.^2,4^ that the literature communicates that 79% of adults use the Internet as a source of health information.^5^ Additionally, 24% of these users access the Internet to obtain food and nutrition information.^6^ One of the most important factors affecting the reliability of food and nutrition information is the credibility of the online source.^7^ Emerging research has examined the influence of health information in social media on health problems such as diabetes, cancer, Alzheimer’s disease, and hypertension among others.^2,8-20^ In the last few years, video-sharing social media has become more popular, with 72% of adults accessing video-sharing social media, including YouTube which is accessed by 63% of adult users.^4^

YouTube (http://www.YouTube.com), first launched in 2005, is among the most utilized social media video sharing platforms on the Internet.^2,21^ Online public communication on YouTube occurs via interactions such as likes, dislikes, and comments to posted videos.^2,7,22^ YouTube also provides content in more than 80 different languages.^23^ YouTube serves as a media channel for promoting education and awareness.^9,16,24-26^ On the other hand, it may provide invalid and misleading information.^7,9^ YouTube videos offer a variety of health information, which is not regulated in terms of the quality of the information provided, nor in the content itself.^27^ Recent studies have reported YouTube videos are a poor source of medical information and have a high probability of propagating misleading information.^2,27^ Consequently, it is necessary that medical, and health information shared on YouTube be reliable to keep from misleading consumers.^6^

The Mediterranean Diet (MedDiet) is the traditional dietary pattern associated with countries surrounding the Mediterranean basin.^28,29^ MedDiet consists of a high intake of fruits, vegetables, legumes, nuts, unrefined whole grains, liberal use of olive oil; moderate-to-high intake of fish; moderate intake of alcohol (primarily wine) through meals; low-to-moderate intake of dairy products; and low intake of red meats, poultry, saturated dietary fat, and sweets.^30-32^ Several studies have shown an inverse association between adherence to MedDiet and oxidative stress, cardiovascular diseases, depression, and several types of cancers.^33-37^ The sheer volume of information available on the Internet has led prior researchers to examine the quality of information communicated.^38^ Not surprisingly, information related to MedDiet found on websites was overwhelmingly poor in quality.^6^

We, therefore, build on prior research examining the potential of YouTube as a resource for health promotion.^39^ The current study bridges an existing gap in the literature on the credibility and quality of information related to MedDiet on online video sharing platforms, specifically YouTube. The overarching purpose of the current review is to assess the quality (credibility and reliability) of MedDiet information available among videos posted online and hosted on YouTube. We also communicate YouTube’s potential for promoting healthy behaviors via MedDiet.

## Methods

The current search and review was conducted in August 2020. Mediterranean diet related videos available on YouTube were systematically searched and examined utilizing standard procedures and DISCERN online video quality evaluation instrument.

Three independent raters searched using the following four search phrases – “Mediterranean Diet; Mediterranean-style Diet; Mediterranean dietary pattern; and Mediterranean Eating Pattern.” Cookies and cache were cleared by each rater prior to conducting search. Prior to abstracting data from search results, all raters checked for consistency of video results. The following criteria were followed in selecting videos for evaluation.

### Inclusion Criteria

Utilizing search terms communicated above, MedDiet video results in the English language only, search periods between January 2010 – August 2020, and 100 top search results were included.

**Exclusion Criteria:** Videos irrelevant to MedDiet keywords, those in non-English language, and hits not in the top 100 ‘highest’ search results were excluded.

Based on previous studies,^2,3,7,10,11,17,19^ YouTube users characteristically only browse through the list presented of the first 60 to 200 videos. We sought to replicate end user experience and evaluate only the first 200 resulting videos fitting inclusion criteria. YouTube settings were adjusted to sort the videos based on the highest view count to the lowest. Video results were excluded if they did not meet the inclusion criteria. A total of 200 videos were collected. Duplicate video results and non-English videos were excluded from analysis. One hundred 41 videos met the criteria for analysis, and each video was evaluated independently by three independent raters from the study team.

The three independent raters are also nutrition experts. Each examined videos independently and collected data accordingly. For data congruency and reproducibility raters followed the same standard procedures and all used the DISCERN evaluation instrument.^38^ The data collected were abstracted using the same objective criteria which included video titles, links, view count, number of comments, number of likes, number of dislikes, and video length. Evaluators classified the speakers featured in the video into 8 categories (Healthcare professional/dietitian/nutritionist, researcher, cook/chef, influencers/actor, personal trainer/coach, not available (N/A), and other). The type of the video was also classified based on three categories (educational, experience (by following MedDiet), and cooking show). The video source was reported based on type of source (for-profit organization, non-profit organization, and individual user). The videos were evaluated using the DISCERN instrument, developed for use in healthcare to judge the quality and reliability of health information.^38^

DISCERN has been used previously in robust peer-reviewed literature,^40-42^ and consists of 16 questions, with each question on a continuous rating scale from 1 to 5 where 1 indicates No; 3 indicates partially and 5 indicates Yes on whether the video fulfills the item’s criteria; 2 and 4 are intermediates on the scale.

DISCERN consists of three categories; A.) evaluation of reliability, dependability, and trustworthiness of video (items 1-8); B.) quality of information presented (items 9-15); and, C.) overall quality of video (item 16). Of note, item 16 is completed separately and its rating scale is distinct to the prior items in the instrument. Item 16s rating scale is continuous from 1 to 5. Where one is defined as a low-quality video with ‘serious shortcomings’; 3 a moderate quality video with ‘some limitations’; and 5 a high quality or video that is a ‘useful source’. For this item, 2 (proximity to low quality) and 4 (proximity to high quality) are intermediates on the scale. To compute a DISCERN score and determine relevant quality level the sum of all 16 items is calculated. A DISCERN score has a total possible maximum score of 80 and minimum score of 16. It is important to note The DISCERN handbook provides little guidance on interpreting total DISCERN scores, and, to date, to our knowledge, no definitive subdivision of the DISCERN score has been formally agreed upon and published.^38,43^ Thus, for the current study we used the following 3 predetermined cut-off points to define video quality level; low quality: 15-37.6, medium quality: 37.7-58.9, and high quality: 59-80.

The reliability of the videos was determined by taking the average of the first 8 questions from (1 to 8), and the quality of the information was assessed based on the average of the questions from 9 to 15. For overall, quality assessed by item 16, the score obtained in the item served as the indicator (as a brief reminder - 1 is defined as a low-quality video with ‘serious shortcomings’; 3 a moderate quality video with ‘some limitations’; and 5 a high quality or video that is a ‘useful source’).

Each video was evaluated independently by 3 content experts. To prevent and reduce the introduction of possible bias, the mean score stemming from each of the 3 evaluations were calculated and recorded for each video.

All analyses were conducted in IBM SPSS version 25. The characteristics of videos (number of views, length in minutes, number of comments, number of likes, and number of dislike) were represented by using mean ±SD as well as minimum and maximum counts. Normal distribution of data was assessed by applying The Kolmogorov-Smirnov test. The associations between the quality of videos using the DISCERN instrument and speaker, type of message and source of information were tested using Chi^2^ due to the data being categorical. Correlation between variables of quality assessments (performed with the DISCERN instrument) and the characteristics of the videos was tested using Spearman’s test as data were not normally distributed. Results were considered significant at *P*-value <.05.

Given the nature of this study, no ethical oversight or approval was found to be necessary and therefore not obtained.

## Results

A total of 141 videos were selected based on inclusion and exclusion criteria and evaluated accordingly. Total number of views per video had a large variability, ranging from 19 to 1 823 994 (Table 1). Number of comments, likes, and dislikes, are also found in Table 1. The shortest video was 15 seconds, while the longest one exceeded one hour. Healthcare professionals represented 22.7% of the speakers in the videos, followed by dietitian/nutritionist at 16.3%. In terms categorization by type of message, the majority of videos (72.3%) were on education.

**Table 1.** Characteristics of Videos on Mediterranean Diet (N = 141).

Variables		
	Mean ± SD	(min - max)
Number of views	68 888.2 ± 170 488.7	(19.06-1,823 994)
Length in minutes	9.7 ± 12.9	(.15-70.26)
Number of comments^a^	152.7 ± 557.4	(0-6038)
Number of “Likes”^b^	1556.3 ± 6550.9	(0-74,000)
Number of “Dislikes”^b^	73.9 ± 263.4	(0-2600)
	**N**	**%**
Speaker		
Healthcare professional	32	22.7
Cook/chef	15	10.6
Researcher	6	4.3
Dietitian/nutritionist	23	16.3
Influencers \ actor	10	7.1
Personal trainer	5	3.5
Other	4	2.8
Not identified	46	32.6
Message type		
Educational	102	72.3
Experience	14	9.9
Cooking show	15	17.7
Source		
Individual user/Layperson	79	56.0
Nonprofit organization	24	17.0
For-profit organization	38	27.0

^a^N = 135.

^b^N = 134.

In regard to quality assessment relevant to the speaker (Table 2); those videos from Cook/Chef speakers had the largest percentage at “Low Total Score” (60% of the videos), while Dietitian/Nutritionist speakers had the largest percentage at “High Total Score” (17.4% of videos). A significant association (*P* = .007) was found for the overall quality score, with healthcare professional/dietitian/nutritionist having the highest percentage of overall medium (50%) and high-quality scores (31.3%). Significant associations were found when comparing the type of the message with the DISCERN scores for reliability (*P* = .006), information quality (*P* = .001) and overall quality (*P* = .000). (Table 3). Cooking videos had the largest percentage of low total quality scores at 68%, compared with 35.7% of experience-based videos (such as visiting the region, or practicing MedDiet to result in specific health outcomes) and 15.7% of educational message videos (*P* = .000). The mean percentage for videos that scored low for information quality was above 50% for all 3 types of message categories, and above 35% for overall quality. Videos scored as medium quality had higher percentages for educational and experience-based messages.

**Table 2.** Quality Assessments of Videos (n = 141) Utilizing DISCERN Instrument Relevant to the Speaker.

Variable	Total Score (15-80)			Reliability			Info. Quality Average			Overall Quality		
	Low N (%)	Medium N (%)	High N (%)	Low N (%)	Medium N (%)	High N (%)	Low N (%)	Medium N (%)	High N (%)	Low N (%)	Medium N (%)	High N (%)
Total	38 (27.0)	88 (62.4)	15 (10.6)	22 (15.6)	87 (61.7)	32 (22.7)	97 (68.8)	40 (28.4)	4 (2.8)	69 (48.9)	39 (27.7)	33 (23.4)
Healthcare professional	2 (6.3)	25 (78.1)	5 (15.6)	3 (9.4)	16 (50)	13 (40.6)	16 (50)	14 (43.7)	2 (6.3)	6 (18.7)	16 (50)	10 (31.3)
Cook/chef	9 (60)	6 (40)	0 (0)	2 (13.3)	13 (86.7)	0 (0)	13 (86.7)	2 (13.3)	0 (0)	12 (80)	2 (13.3)	1 (6.7)
Researcher	1 (16.7)	5 (83.3)	0 (0)	0 (0)	5 (83.3)	1 (16.7)	3 (50)	3 (50)	0 (0)	5 (83.3)	1 (16.7)	0 (0)
Dietitian/nutritionist	3 (13)	16 (69.6)	4 (17.4)	3 (13)	16 (69.6)	4 (17.4)	15 (65.2)	6 (26.1)	2 (8.7)	10 (43.5)	4 (17.4)	9 (39.1)
Influencers \ actor	4 (40)	5 (50)	1 (10)	2 (20)	6 (60)	2 (20)	9 (90)	1 (10)	0 (0)	8 (80)	1 (10)	1 (10)
Personal trainer	2 (40)	3 (60)	0 (0)	1 (20)	2 (40)	2 (40)	4 (80)	1 (20)	0 (0)	2 (40)	1 (20)	2 (40)
Other	1 (25)	3 (75)	0 (0)	0 (0)	3 (75)	1 (25)	4 (100)	0 (0)	0 (0)	2 (50)	2 (50)	0 (0)
Not identified	16 (34.8)	25 (54.3)	5 (10.9)	11 (23.9)	26 (56.5)	9 (19.6)	33 (71.7)	13 (28.3)	0 (0)	24 (52.2)	12 (26.1)	10 (21.7)
***P*-value**	.082			.321			.154			.007		

Data are presented as number of videos and (%).

*P*-value is calculated using Chi^2^ test.

**Table 3.** Quality Assessments of Videos (n = 141) Utilizing DISCERN Instrument Relevant to Message Type.

Variable	Total Score (15-80)			Reliability			Info. Quality Average			Overall Quality		
	Low N (%)	Medium N (%)	High N (%)	Low N (%)	Medium N (%)	High N (%)	Low N (%)	Medium N (%)	High N (%)	Low N (%)	Medium N (%)	High N (%)
Total	38 (27.0)	88 (62.4)	15 (10.6)	22 (15.6)	87 (61.7)	32 (22.7)	87 (61.7)	40 (28.4)	4 (2.8)	69 (48.9)	39 (27.7)	33 (23.4)
Educational	16 (15.7)	71 (69.6)	15 (14.7)	16 (15.7)	55 (53.9)	31 (30.4)	60 (58.8)	38 (37.3)	4 (3.9)	36 (35.3)	34 (33.3)	32 (31.4)
Experience	5 (35.7)	9 (64.3)	0 (0)	3 (21.4)	10 (71.4)	1 (7.1)	12 (85.7)	2 (14.3)	0 (0)	9 (64.3)	4 (28.6)	1 (7.1)
Cooking show	17 (68)	8 (32)	0 (0)	3 (12)	22 (88)	0 (0)	25 (100)	0 (0)	0 (0)	24 (96)	1 (4)	0 (0)
***P*-value**	.000			.006			.001			.000		

Data are presented as number of videos and (%).

*P*-value is calculated using Chi^2^ test.

Table 4 communicates quality assessment of videos relevant to information source. There was a significant association between the total score (*P* = .012), reliability (*P* = .005) and information quality (*P* = .026) of the videos and information source (individual user/layperson, Nonprofit organization, For-profit organization), but no significant association between type of information source and overall video quality was found (*P* = .162). The highest percent of reliability and overall quality scoring were obtained for videos with Nonprofit organizations as the source (54.2% and 41.7%, respectively). Individual user/layperson videos had the largest percentage of low information quality average scores (74.7%), followed closely by for-profit video sources (71.1%) presenting information measured by DISCERN as low information quality (Table 4).

**Table 4.** Quality Assessments of Videos (n = 141) Utilizing DISCERN Instrument Relevant to Information Source.

Variable	Total Score (15-80)			Reliability			Info. Quality Average			Overall Quality		
	Low N (%)	Medium N (%)	High N (%)	Low N (%)	Medium N (%)	High N (%)	Low N (%)	Medium N (%)	High N (%)	Low N (%)	Medium N (%)	High N (%)
Total	38 (27.0)	88 (62.4)	15 (10.6)	22 (15.6)	87 (61.7)	32 (22.7)	97 (68.8)	40 (28.4)	4 (2.8)	69 (48.9)	39 (27.7)	33 (23.4)
Individual user/Layperson	24 (30.4)	50 (63.3)	5 (6.3)	14 (17.7)	52 (65.8)	13 (16.5)	59 (74.7)	20 (25.3)	0 (0)	44 (55.7)	19 (24.1)	16 (20.3)
Nonprofit organization	4 (16.7)	12 (50)	8 (33.3)	3 (12.5)	8 (33.3)	13 (54.2)	11 (45.8)	10 (41.7)	3 (12.5)	6 (25)	8 (33.3)	10 (41.7)
For-profit organization	10 (26.3)	26 (68.4)	2 (5.3)	5 (13.2)	27 (71)	6 (15.8)	27 (71.1)	10 (26.3)	1 (2.6)	19 (50)	12 (31.6)	7 (18.4)
***P*-value**	.012			.005			.026			.162		

*P*-value is calculated using Chi^2^ test.

Preliminary correlations video characteristics and quality are summarized in Table 5. There was a weak but significant (rho = −.170, *P* < .05) negative correlation between overall quality of the video and number of dislikes. Exploratory analyses did not find other statistically significant correlations.

**Table 5.** Correlation (Spearman’s rho) Between Quality Assessment Variables (Utilizing DISCERN instrument) and Video Characteristics.

	Reliability	Info. Quality Average	Overall Quality	Total Score	Number of Likes	Number of Dislikes	Number of Comments	Number of Views
Reliability	1.000	.488^**^	.571^**^	.632^**^	.077	−.029	.034	−.109
Info. quality average	—	1.000	.624^**^	.576^**^	.096	.046	.097	.066
Overall quality	—	—	1.000	.624^**^	−.042	−.170^*^	−.053	−.097
Total score	—	—	—	1.000	.014	−.063	.061	−.014
Number of likes	—	—	—	—	1.000	.853^**^	.926^**^	.758^**^
Number of dislikes	—	—	—	—	—	1.000	.859^**^	.768^**^
Number of comments	—	—	—	—	—	—	1.000	.770^**^
Number of views	—	—	—	—	—	—	—	1.000

Correlation was assessed using Spearman’s test.

*P-value<.05, **P-value<.01.

## Discussion

This research confirms both the variability in the quality of information provided on the Mediterranean diet and related health benefits in YouTube videos as rated by content experts researchers in the field,^6^ as well as the lack of correlation between the health information quality of these videos and consumer response to said videos. Because of the unregulated nature of this forum, it is not surprising that there was little correlation between the researcher ratings of the videos using DISCERN and the video characteristics evaluated. The present research indicates that the number of views, likes, dislikes, and comments is not associated with, and should not be used as an indicator for quality of videos on the topics of MedDiet, and health; this mirrors prior findings among published studies on health information found on YouTube.^10,44^ These findings further communicate an area for concern for all health practitioners, as the videos that were assessed as lower quality were not correlated with consumer interactions which can result in amplifying the message of a video such as likes and views. While the significant yet weak negative correlation between overall video quality and the number of dislikes could be a promising trend, there is no indication that the number of ‘dislikes’ is due to the veracity of the information presented. eHealth literacy is defined as the ability to seek, find, understand, and appraise health information from electronic sources, and apply the knowledge gained to addressing or solving a health problem.^45^ The findings highlight a need to increase consumer literacy about the reliability of video-based information in online forums such as YouTube. The researchers assessed MedDiet videos utilizing the DISCERN tool to determine overall video quality, with significant associations found between the type of professional presenting the information, however, the scores for reliability, information quality, and the total score were not significantly associated with the profession of the presenter. Because of the increasing role of the Internet and volume of healthcare information provided on platforms such as YouTube^18,46^ it is imperative that quality be a concern of professionals providing information in this format.^47-49^ While there was a significant association between MedDiet video source and DISCERN total, information quality, and reliability scores and it was determined that non-profit funded sources had the highest quality ratings compared to for-profit, and individual sources. In general, the distribution of quality scores was relatively wide and poorly correlated with the video characteristics. This finding is similar to other content analyses that have found a mixed quality of health information online and a lack of well-established guidelines for professionals on the best methods to deliver evidence-based information to consumers via the Internet.^10,50,51^

In reflecting on the current study’s findings on MedDiet, it is of interest how our results compare to videos for other health conditions. We acknowledge that further research is necessary to speak on findings relevant to multiple health conditions. Yet, in similar prior efforts, such as those by Stellefson et al,^14^ on YouTube’s potential to reach and educate COPD patients, findings are astonishingly similar in the need to encourage researchers and practitioners to lead efforts on creating high quality educational materials respective to their field of expertise.^14^

This study has some limitations. Selection of the videos was made using a limited number of key words; hence, some videos could have been missed. Additionally, YouTube is known to be a constantly changing platform which implies that the number of comments, likes and dislikes could change. Finally, only videos in English were evaluated, meaning videos in other languages could influence the findings of the current study.

Information on YouTube regarding MedDiet and health, as with other health information available on the platform, is varied in quality, and more work is needed to improve the quality of information provided in videos made on this topic. The highest quality videos reviewed were attributed to healthcare professional/dietitian/nutritionist; and, were categorized as educational, and provided by non-profit sources. The number of a video’s views, comments, likes and dislikes is not correlated with, and subsequently should not be used as a gauge for quality. Based on these findings, it is recommended that healthcare professional/dietitian/nutritionist, researchers, universities, and governmental and non-profit organizations should be encouraged to create evidence-based content that is of interest to viewers to help balance what clients, and their families may access on popular media sites and the Internet. Additionally, our findings speak to the need for eHealth literacy training to increase the public’s ability to seek, find, understand, and appraise health information from electronic sources and apply the knowledge gained to addressing or solving a health problem.

**So What?**

**What is Already Known on this Topic?**

The Mediterranean diet (MedDiet) is internationally recognized as an antiobesogenic dietary model. Social media is considered a highly important tool for communicating health information and serves as a channel for promoting education and awareness.

**What does this Article Add?**

This article assesses the quality of MedDiet information available on YouTube ^TM^. This article also communicates YouTube’s potential for promoting healthy behaviors via MedDiet, thereby filling a gap in the scholarly literature with regards to the MedDiet’s visibility, credibility, and how information on this dietary pattern is portrayed on social media.

**What are the Implications for Health Promotion Practice or Research?**

Information on YouTube regarding MedDiet and health promotion, as with other health information available on the platform, is varied in quality, and more work is warranted to improve the quality of information provided in videos made on this topic. Findings also speak to the necessity for eHealth literacy training to increase the public’s ability to seek, find, understand, appraise, and apply health information from electronic sources.
